# The categorisation of the Short Warwick Edinburgh Mental Wellbeing Scale scores: an exploration from an epidemiological perspective

**DOI:** 10.3389/fpsyt.2025.1674009

**Published:** 2025-11-28

**Authors:** Lawrence T. Lam, Mary K. Lam

**Affiliations:** 1 Faculty of Medicine, Macau University of Science and Technology, Macao, Macao SAR, China; 2 Faculty of Medicine and Health, The University of Sydney, Sydney, NSW, Australia; 3 Faculty of Health, University of Technology Sydney, Sydney, NSW, Australia; 4 School of Health and Biomedical Sciences, RMIT University, Bundoora, Melbourne, VIC, Australia

**Keywords:** SWEMWBS, agreement, sensitivity, specificity, receiver operating characteristic-area under the curve ROC AUC

## Abstract

**Background:**

There has been a notable increase in the utilisation of SWEMWBS as a measure of mental well-being globally. To enhance its interpretability for both healthcare professionals and laypeople, categorising SWEMWBS scores is considered beneficial. Two approaches have been recommended, yet they have not been thoroughly investigated. This study aimed to explore the categorisation of the scores from an epidemiological perspective.

**Methods:**

Adopting PHQ-9 results, as suggested by the original scale author, to be the benchmarking comparator and employing the epidemiological approach, the concordance between SWEMWBS and PHQ-9 was examined using data from a health survey. The scales were categorised following the recommended cutoffs suggested by the authors. An additional cutoff was generated from the Nonparametric Receiver Operating Characteristic (ROC) Analysis and verified using the multiclass ROC analysis. The agreement indicators, including the sensitivity, specificity, Positive Predictive Value, Negative Predictive Value, Likelihood Ratio Positive, Likelihood Ratio Negative, and Receiver Operating Characteristic-Area Under the Curve (ROC AUC), were calculated.

**Results:**

The categorisation of SWEMWBS scores by benchmarking yielded the highest sensitivity, but the smallest specificity with 86.1% (95% C.I. = 84.1%-87.9%) and 56.6% (95% C.I. = 49.1%- 63.9%) respectively. Categorisation using the mean and SD approach resulted in a sensitivity of 81.3% (95% C.I. = 79.1%- 83.4%) and a specificity of 68.1% (95% C.I. = 60.8%-74.8%). In contrast, categorisation using the ROC analysis approach provided a sensitivity of 76.5% (95% C.I. = 73.8%-79.0%) and a specificity of 77.5% (95% C.I. = 70.7%-83.3%). The ROC AUC values were moderately low with the largest being 0.769 (95% C.I. = 0.737-0.802).

**Conclusions:**

The concordance of the Chinese version of the SWEMWBS has been examined using PHQ-9 as the benchmarking comparator. The results indicate moderate sensitivity, specificity, LR+, and LR- values.

## Background

The Warwick-Edinburgh Mental Well-Being Scale (WEMWBS) and its 7-item Short Form (SWEMWBS) are among the few psychometric instruments specifically designed to assess mental well-being (1). Since the validation of SWEMWBS (2, 3), there has been a notable increase in the translation, validation, and utilisation of SWEMWBS as a measure of mental well-being globally (4–13). To facilitate the use of the scale and enhance its interpretability for both healthcare professionals and laypeople, categorising SWEMWBS scores is considered beneficial (14).

According to information provided by Warwick University, scores can be categorised using statistical and benchmarking approaches (14). The statistical approach is based on the observed characteristic that the scores follow an approximately normal distribution. Guided by the principles of probability theory, the probability density function (PDF) of a normal distribution *N*(0, *σ*^2^) encompasses approximately 68.3% of the area under the curve within one standard deviation (SD) from the mean, both above and below (15). The remaining area under the curve is evenly distributed in the upper and lower tails, with 15.85% each. Based on this concept, cutoff points are suggested to be one SD above and below the mean value, with approximately 15% classified as high/good well-being and 15% classified as low/poor well-being (14). Given a mean value of 23.5, an SD of 3.9, with the possible scores ranged between 7 and 35, found in the UK general population sample (2), it was suggested that scores of 27.5 and 19.5 serve as cutoff points for high/good and low/poor well-being, respectively (14). Benchmarking is a simpler approach based on the high correlation between SWEMWBS scores and the Primary Care Evaluation of Mental Disorders Patient Health Questionnaire (PHQ-9) (16). Consequently, SWEMWBS scores are suggested to be benchmarked with the cutoff points of PHQ-9 at 5 and 10, with a SWEMWBS score <18 indicating probable clinical depression and 19–20 indicating possible clinical depression (14). While these approaches have facilitated data analysis and, to some extent, simplified the interpretation of raw and transformed metric scores, further evidence is required to support the utilisation of the categorised scoring in clinically oriented environments, such as mental health services.

An essential type of study in epidemiology is measurement investigation (17). The primary objective of measurement studies is to determine the accuracy and validity of a test or instrument, providing evidence for its utility in an appropriate setting (18). To determine the accuracy of an instrument, such as a psychometric scale, the core of the measurement test study involves comparing the scale under investigation with a well-established comparator (17). Various statistical approaches can be employed to determine the degree of agreement between the instrument under investigation and the comparator, reflecting the accuracy of the studied scale (18). So far, few studies have been found in the literature investigating the properties of SWEMWBS from an epidemiological perspective (2).

Given the growing utilisation of the SWEMWBS in various settings globally, it is prudent to explore the psychometric properties of the scale from an epidemiological perspective. Based on the aforementioned recommended methods of scoring categorisation by Warwick University, this exploratory study aimed to examine the performance of these cutoff scores as to how closely they can align with the categorisation of the PHQ-9 adopted as the benchmarking comparator.

## Methods

### Sample and data collection

The data for this study was gathered through a population-based cross-sectional health survey, utilising a self-reported online questionnaire. Conducted between April and July 2024, the survey targeted adult residents of Macau. The questionnaire was disseminated via 23 associations and societies that collaborated in the city-wide study, including professional bodies, community associations, and non-government organisations. With the support of these organisations’ management, members were encouraged to participate through public appeals and personal invitations. The potential participant pool exceeded 50,000 individuals, representing nearly 9% of Macau’s adult population. The sample comprised 1,460 respondents, of which 1,001 were females (68.6%), and about 45% fell within the 18–34 age group (n=655, 44.9%). Ethics approval was obtained from the Faculty Ethics Research Committee of the Faculty of Medicine, Macau University of Science and Technology (MUST-FMD-200402025001).

### Measurements

#### The Short Form of the Warwick-Edinburgh Mental Well-being Scale

The 14-item WEMWBS has been validated and is widely utilised in numerous mental well-being studies, demonstrating robust content and structural validity, with a single factor confirmed by Confirmatory Factor Analysis (1). It also exhibits high reliability, with Cronbach’s alpha scores ranging from 0.89 to 0.91. The WEMWBS demonstrates strong correlations with other mental health and well-being scales and lower correlations with scales measuring overall health, with a test-retest reliability of 0.83 at one week (1). The Short Warwick-Edinburgh Mental Well-being Scale (SWEMWBS), derived from the original scale with 7 items by Stewart-Brown et al. in 2009, employs a 5-point frequency Likert response set ranging from 1=rarely to 5=all the time, resulting in a total score range of 7 to 35. More information on the SWEMWBS can be found on the official website (14). The Chinese version of the SWEMWBS, first translated by Ng et al. in 2014, has been used in several studies. A few validation studies employed the classical test theory approach, all using samples from a single location, primarily Hong Kong, except one conducted in China (7, 10, 19–24). Overall, these studies indicated that the scale has good validity and reliability for use in the Chinese population (7, 10). The utilisation of the Chinese version of the instrument, in both traditional and simplified characters, in various studies confirmed that the high psychometric properties of the scale. In this study, the raw scores of the scale were used for the analyses.

### Benchmarking comparator

In this study, the Primary Care Evaluation of Mental Disorders Patient Health Questionnaire (PRIME-MD-PHQ) (25) was employed as the benchmarking comparator due to its use by the original authors for cutoff recommendations (14). The reason for adopting PHQ as the quasi comparator, apart from the aforementioned recommendation of the scale authors, was that there is no commonly recognised gold standard of mental well-being. In examining the utility of SWEMWBS, it would be prudent to use a sensitive instrument for assessing common mental disorders as a proxy benchmarking standard. The PHQ-9 is extensively utilised for evaluating mental health issues, particularly depression, among outpatients and the general population (25). This unidimensional scale consists of nine items designed to assess symptoms of depression. Respondents are asked to reflect on their experiences over the past two weeks using a 4-point frequency Likert scale, ranging from 0 (Not at all) to 3 (Nearly every day). An example item is “Feeling down, depressed, and hopeless.” The instrument has undergone validation and standardisation in numerous studies across various countries (25–27). This study will utilise the nine-item version (PHQ-9). The Chinese version of the PHQ-9 has been translated, and validated, and is widely used in Chinese-speaking countries (28). It demonstrates reliability, with an internal consistency Cronbach’s alpha of 0.86 and a two-week test-retest correlation of 0.86. The scale exhibits good convergent validity, with a positive correlation of 0.29 (p<0.001) with the SDS and a negative correlation of -0.47 with the SF-36. The area under the ROC curve is 0.92 (95% confidence interval: 0.86–0.97). A cutoff score of seven or higher on the PHQ-9 has a sensitivity of 0.86 and a specificity of 0.86 (28). For the categorisation of scores, the authors recommended cutoffs of 5, 10, 15, and 20 for mild, moderate, moderately severe, and severe depression, respectively (29).

### Data management and analysis

#### Data management

Data were managed and analysed using STATA statistical software (StataNow 19.0). The categorisation of the SWEMWBS and PHQ-9 scores was conducted following the recommended cutoffs. Hence, the SWEMWBS scores were categorised into three groups by the mean with standard deviation and the benchmarking approaches. Adopting the original meaning suggested by the authors, these groups were labelled as poor, moderate, and well/normal. For the PHQ-9, the scores were categorised into five groups namely normal, mild, moderate, moderately severe, and severe depression. To synchronise the number of groups in both scales, the five groups of PHQ-9 were then regrouped into three normal/mild, moderate, and moderately severe/severe. The two scales were negatively correlated suggesting the response sets were in reversed directions. For ease of data analysis, SWEMWBS scores were reversely coded so that the direction of both scales was unified with a higher-ordered group having a higher risk of poor mental health/wellbeing. In terms of the categorisation of the scale, groups were first generated from the original raw scores following the two categorisation approaches. Then the order of the groups was reversely coded so that the direction of the groups synchronised with the direction of the PHQ-9 (normal/mild, moderate, severe).

#### Data analysis

Descriptive statistics of the demographic variables, SWEMWBS, and PHQ-9 were generated as means and standard deviations for continuous variables and frequencies and percentages for categorical variables. The values and the 95% Confidence Intervals (95% C.I.) of the concordance or agreement indicators between categorised SWEMWBS, based on the two approaches, and PHQ-9 as the benchmarking comparator were calculated with the severe group as the positive case group. These included the accuracy, sensitivity, specificity, positive predictive value (PPV), negative predictive value (NPV), positive likelihood ratio (LR+), negative likelihood ratio (LR-), and the Receiver Operating Characteristic Area Under the Curve (ROC AUC). To further explore the “cutoff” point of the SWEMWBS for probable cases empirically, the reversed scores were subjected to a Nonparametric ROC Analysis using the same benchmarking comparator and positive case criteria as the two approaches. Once the “cutoff” point of SWEMWBS was identified, it was then used to calculate the values and 95% C.I. of the agreement indicators. For determining the “cutoff” point, the principles regarding the sensitivity and specificity propounded by Power et al. were applied (30). It was emphasised that, for a test to be useful, the sum of the sensitivity and specificity should be 1.5 or 150% (30). The rationale for such criteria was that the true positive and the true negative rates of the test should be at least 75% each with some variations in both rates. To verify the results obtained from the empirical “cutoff”, the multiclass ROC analysis was applied to the data controlling for demographic variables. A type I error rate of 5% was used for all hypothesis tests.

## Results

### Descriptive statistics

The sample included 1,460 participants, as shown in **Table 1**. The majority were female (n=1001, 68.6%) and younger, with nearly 45% aged between 18 and 34 years (n=655, 44.9%). Slightly less than half were either married or in a de facto relationship (n=693, 47.5%), and two-thirds had achieved a university-level education or higher (n=966, 66.2%). A total of 672 participants (46%) identified as caregivers. The mean score on the Chinese SWEMWBS was 21.5 (SD = 4.9) out of a possible 35. Of these, 363 (24.9%) were in the lowest 15% of the sample and 281 (19.3%) could be categorised as having probable clinical depression based on the two approaches. For depression, the average scores were 6.9 (SD = 6.0) with 182 (12.5%) classified as severe and very severe depression.

**Table 1:** Descriptive information of the demographics, SWEMWBS, and PHQ-9 of the sample (N = 1460).

Variables	Frequency (%) or Mean (SD)
Demographics	
Sex	
Male	459 (31.4%)
Female	1001 (68.6%)
Age group (years)	
18-34	655 (44.9%)
35-54	589 (40.3%)
55 +	216 (14.8%)
Marital status	
Married/De facto	693 (47.5%)
Others	767 (52.5%)
Educational level	
University or higher	966 (66.2%)
Others	494 (33.8%)
Main study variables	
SWEMWBS categorised by mean (s.d.)	
Poor (lowest 15%)	363 (24.9%)
Moderate	748 (51.2%)
Good (highest 15%)	349 (23.9%)
SWEMWBS categorised by benchmarking)	
Probable clinical depression	281 (19.3%)
Possible clinical depression	162 (11.1%)
Mentally well	1017 (69.6%)
SWEMWBS score	23.2 (5.8)
Gold standard	
PHQ-9 categorised	
Severe/Very Severe	182 (12.5%)
Moderately	209 (14.3%)
Mild/Normal	1069 (73.2%)
PHQ-9 score	6.9 (5.9)

### Agreement analysis

**Figure 1:**
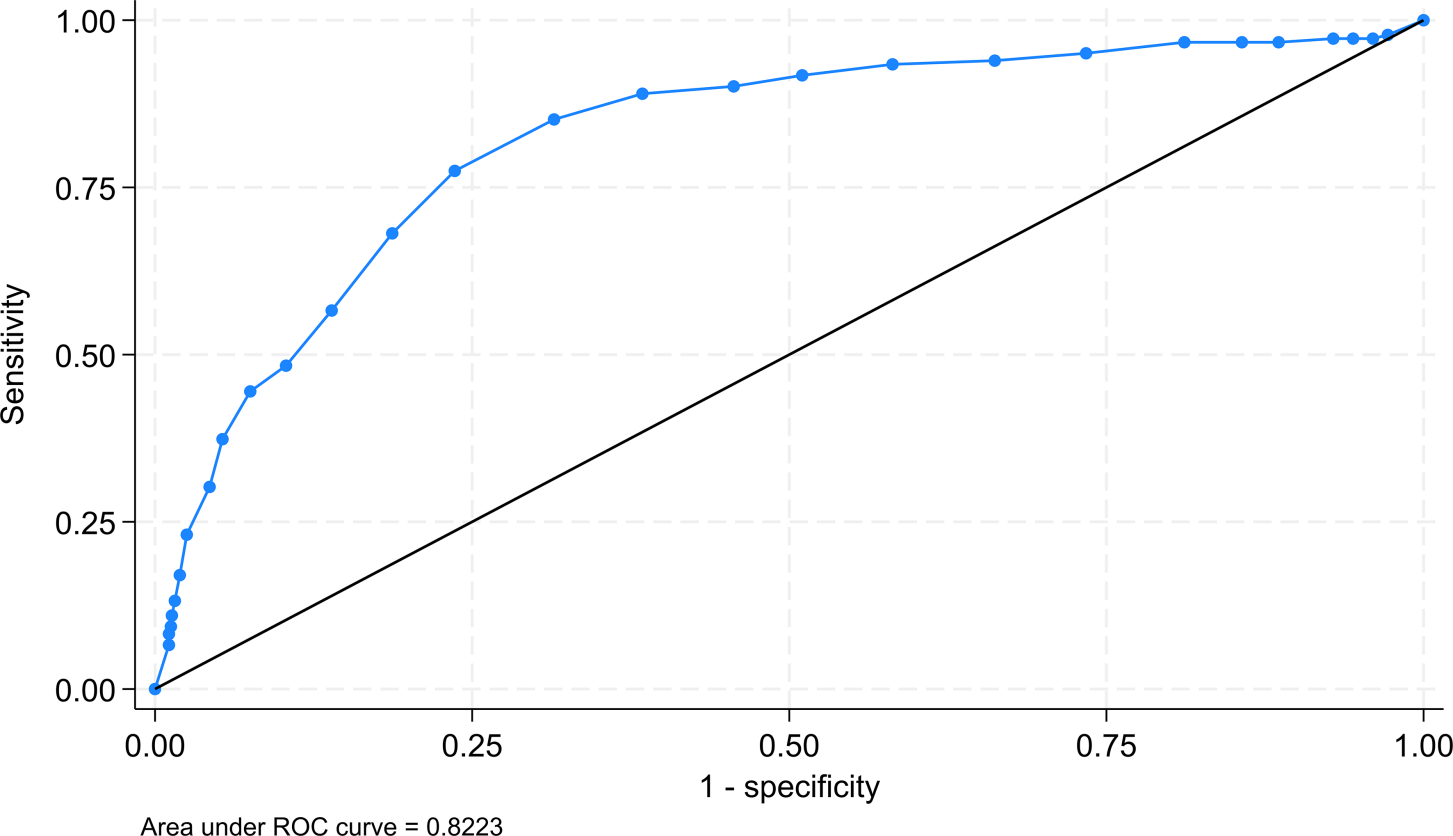
ROC of using the SWEMWBS raw score for determining the cutoff for probable positive cases.

The ROC analysis, using the same “gold” standard and positive diagnosis criteria as the two approaches, resulted in a cutoff point of 22 which provided the highest sensitivity and specificity sum value of 153.9% (**Figure 1**). This cutoff score corresponded to the score of 20 on the original scale.

**Figure 2:**
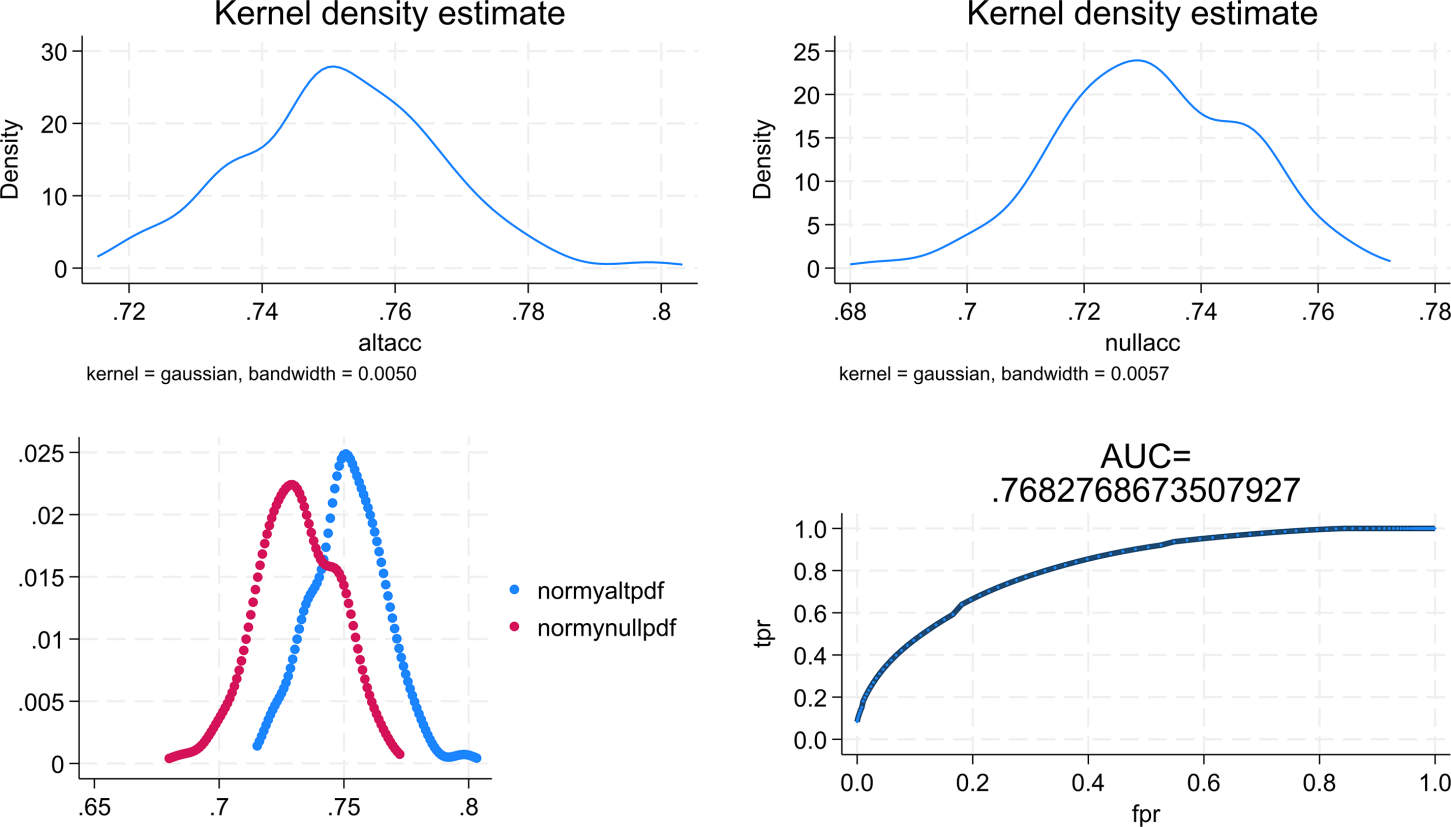
Graphical results obtained from the multiclass ROC analysis. altacc, alternative accuracy; nullacc, null accuracy; mormy nullpdf =estimated probability density function for the null class; normy altpdf, estimated probability density function for the alternative class; tpr, true probability; fpr, false probability.

The results of the agreement indicators are summarised in **Table 2**. As shown, the categorisation of SWEMWBS scores by benchmarking yielded the highest sensitivity, but the smallest specificity with 86.1% (95% C.I. = 84.1%-87.9%) and 56.6% (95% C.I. = 49.1%- 63.9%) respectively. Categorisation using the mean and SD approach resulted in a sensitivity of 81.3% (79.1%- 83.4%) and a specificity of 68.1% (95% C.I. = 60.8%-74.8%). In contrast, categorisation using the ROC analysis approach provided a sensitivity of 77.5% (95% C.I. = 70.7%-83.3%) and a specificity of 76.4% (95% C.I. = 73.9%-78.7%). As expected, the categorisation based on the benchmarking approach provided the largest LR+ value of 4.06 (95% C.I. = 3.37-4.90), but also a large LR- of 0.504 (95% C.I. = 0.427-0.596). On the other hand, categorisation based on ROC analysis had a smaller LR+ value of 3.28 (95% C.I. = 2.89-3.72), but also a smaller LR- value of 0.295 (95% C.I. = 0.225-0.387). In terms of the ROC AUC, the ROC analysis cutoff yielded the largest value of 0.769 (95% C.I. = 0.737-0.802) in comparison to the other two approaches (**Table 2**). This result suggested the ability of the scores of SWEMWBS in classifying depressive cases based on the definition of PHQ-9 was only moderate. The data were then subjected to the multiclass ROC Analysis by fitting the multinomial logistic regression model. The aforementioned results were also supported by the multiclass ROC analysis resulting in an overall estimated ROC AUC of 0.768 (**Figure 2**).

**Table 2:** Results of the agreement indicators for SWEMWBS measured against the gold standard for probable clinical depression.

Indicators	Categorised by mean and SD	Categorised by benchmarking	Categorised based on ROC analysis cutoff at 20
Accuracy	79.7% (77.1%-82.1%)^a^	82.4% (79.9%-84.6%)	76.5% (73.8%-79.0%)
Sensitivity	81.3% (79.1%- 83.4%)	86.1% (84.1%-87.9%)	77.5% (70.7%-83.3%)
Specificity	68.1% (60.8%-74.8%)	56.6% (49.1%- 63.9%)	76.4% (73.9%-78.7%)
PPV	34.2% (29.3%-39.3%)	36.7% (31%-42.6%)	31.8% (27.5%-36.4%)
NPV	94.7% (93.2%-96%)	93.3% (91.7%-94.7%)	96% (94.6%-97.1%)
LR+	3.64 (3.13-4.24)	4.06 (3.37-4.90)	3.28 (2.89-3.72)
LR-	0.392 (0.316-0.486)	0.504 (0.427-0.596)	0.295 (0.225-0.387)
ROC AUC	0.747 (0.712-0.783)	0.713 (0.676-0.751)	0.769 (0.737-0.802)

a 95% C.I. PPV, Positive Predictive Value; NPV, Negative Predictive Value; LR+, Likelihood Ratio +ve LR- , Likelihood Ratio -ve; ROC AUC, Receiver Operating Characterisitc Area Under the Curve.

## Discussion

The SWEMWBS was initially designed to provide a theoretically driven and validated means for evaluating mental well-being, a concept that reflects the positive aspects of mental health (31–33). The increasing utilisation of the SWEMWBS as a mental health-related measure calls for more studies from an epidemiological perspective (34, 35). However, the scarcity of studies in the literature indicates a significant knowledge gap in the field of mental well-being research. Consequently, the author is motivated to further explore the scale’s psychometric properties from the epidemiological perspective, focusing on the issue of accuracy while adopting PHQ-9 as the benchmarking comparator.

The results suggested that both recommended approaches for categorising scale scores yielded a sensitivity of greater than 80% (86.1% for benchmarking and 81.3% for using mean and standard deviation). Conversely, the specificity was much weaker, with only 56.6% and 68.1% for the benchmarking and mean with standard deviation approaches, respectively. Using the cutoff provided by the ROC analysis resulted in a sensitivity of 77.5% and a specificity of 76.4%. The ROC AUC of all three approaches were also moderate. As such, these results did not differ significantly, suggesting no single approach for categorisation is superior to the others. This suggested that SWEMWBS would be better utilised as a screening measure than other purposes. Given the absence of comparable studies in the existing literature, a comparison of results is shown to be difficult. The results obtained are considered to be novel and unique.

There could be various reasons for the results obtained. One possible reason is the use of PHQ-9 as the comparator on which the benchmarking approach has been based. PHQ-9 is a highly sensitive instrument with good psychometric properties for assessing depression in both clinical settings and the general population (29). However, the instrument was designed for assessing depression, and the underlying construct that the items attempt to capture is clinical depression reflected through the symptoms described in the items. On the other hand, SWEMWBS aims to evaluate mental well-being, with the underlying constructs involving two different dimensions: the hedonic and eudaimonic aspects (36). The hedonic aspect refers to the individual subjective feeling of happiness and satisfaction in life, whereas the eudaimonic aspect is related to psychological functioning and the actualization of the individual’s potential, capacity, and positive relationship with self and others (36). There are similarities between these two scales, particularly the hedonic domain of the SWEMWBS, since both consist of items on happiness, personal satisfaction in life, and psychological functioning. However, they differ on the eudaimonic aspect that PHQ-9 does not cover much. While these constructs are highly and significantly correlated with depression, they do not assess the same phenomena. Therefore, there arises a question as to whether PHQ-9 could be used as a “gold” standard for benchmarking or examining the accuracy of SWEMWBS. This calls for further studies on the accuracy of the scale using other appropriate instruments as the “gold” standard. Unfortunately, as aforementioned, there is no commonly recognised gold standard of mental well-being so far. As such, a possible solution is to use a set of well-validated instruments as comparators for different aspects of SWEMWBS. Another possible reason is sampling biases. As aforementioned, the majority, nearly 69%, of the respondents were females, and nearly 45% were in the 18–34 year group. The sex and age imbalance characteristics might have affected the respondents’ responses to the two scales. It has been demonstrated that the overall prevalence of depression in males is lower than that of females, with 6.6% and 9.3%, respectively, before the COVID-19 pandemic (37). On the other hand, the performance of the SWEMWBS as a scale has been demonstrated to be better in males than in females (38). This might help explain the issue of moderate sensitivity and specificity. Further research is warranted to explore the sex and age-specific accuracy of SWEMWBS.

## Conclusions

In conclusion, the psychometric properties of the Chinese version of the SWEMWBS have been further examined using the epidemiological approach, specifically the concordance with PHQ-9 as the benchmarking comparator. The results indicate moderate sensitivity and specificity without outstanding LR+ and LR- values. It is noteworthy that SWEMWBS is a validated instrument of assessing mental well-being, not depression. Hence, caution should be taken when applying the results as an indicator of mental health.

## Data Availability

The raw data supporting the conclusion of this article is not publicly available a protect participant confidentiality and privacy. Requests to access the datasets should be directed to the corresponding author.
